# Lay provider HIV testing: A promising strategy to reach the undiagnosed key populations in Vietnam

**DOI:** 10.1371/journal.pone.0210063

**Published:** 2018-12-31

**Authors:** Bao Ngoc Vu, Kimberly Elizabeth Green, Huong Thi Thu Phan, Minh Hung Tran, Huu Van Ngo, Son Hai Vo, Trang Minh Ngo, Anh Hong Doan, An Bao, Linh Hong Dang, Giang Thi Tra Ha

**Affiliations:** 1 Mekong Regional Program, PATH, Hanoi, Vietnam; 2 Vietnam Administration of HIV/AIDS Control, Ministry of Health, Hanoi, Vietnam; 3 Center for Creative Initiatives in Health and Population, Hanoi, Vietnam; 4 United States Agency for International Development, Hanoi, Vietnam; University of Miami, UNITED STATES

## Abstract

**Background:**

In Vietnam, reaching the remaining one-third of undiagnosed people living with HIV and facilitating their antiretroviral therapy (ART) enrollment requires breakthrough approaches. We piloted lay provider HIV testing as an innovative approach to reach at-risk populations that never or infrequently HIV test at facility-based services.

**Methods:**

We conducted a cross-sectional survey and analysis of routine program data in two urban provinces (Hanoi and Ho Chi Minh City) and two rural mountainous provinces (Nghe An and Dien Bien) from October 2015 through September 2017. Acceptability of lay provider testing was defined as the proportion of first-time HIV testers utilizing the service, and effectiveness was measured by HIV positivity and ART initiation rates. Univariate and multivariate analyses were used to determine lay provider testing preference and factors associated with that preference.

**Results:**

Among 1,230 individuals recruited for face-to-face interviews, 74% belonged to key populations: people who inject drugs accounted for 31.4%; men who have sex with men, 60.4%; and female sex workers, 8.2%. Most clients (67%) reported being first-time HIV testers, and the majority (85.8%) preferred lay provider testing to facility-based testing. Multivariate analysis found that clients in urban areas (adjusted odds ratio [aOR] = 2.50; 95% confidence interval [CI]: 1.30–4.90) and those who had a university or higher education (aOR = 1.83; 95% CI: 1.05–3.20) were more likely to prefer lay provider testing. Lay provider testing yielded a higher HIV positivity rate (4.1%), particularly among first-time testers (6.8%), compared to facility-based testing (nationally estimated at 1.6% in 2016) and had a high ART initiation rate (91%).

**Conclusions:**

Our findings suggest that lay provider HIV testing is an effective approach to reach previously unreached at-risk populations, and, therefore, a critical addition to accelerating Vietnam’s attainment of the Joint United Nations Programme on HIV/AIDS 90-90-90 goals.

## Introduction

Reaching people who are HIV undiagnosed is critical to achieving the first “90” of the Joint United Nations Programme on HIV/AIDS 90-90-90 global HIV targets by 2020 (i.e., to diagnose 90% of people with HIV globally) [1]. Achieving this goal requires new approaches, new strategies, and new models of HIV testing services tailored to different epidemic contexts [2].

Vietnam was the first country in Asia to commit to the global target of “90-90-90 by 2020” and the goal of “ending AIDS by 2030” [3]. The HIV epidemic in Vietnam is concentrated among four key populations (KPs): people who inject drugs (PWID), men who have sex with men (MSM), transgender women (TGW), and female sex workers (FSW). Vietnam has an estimated 250,000 people living with HIV (PLHIV), but as of 2016, only 170,000 were diagnosed and 116,000 were on antiretroviral therapy (ART) [4].

Annual HIV testing uptake among KPs in Vietnam continues to be exceptionally low. Before 2016, only one-third of PWID, MSM, and FSW tested annually for HIV and received their test results [5]. The positivity rate resulting from facility-based HIV testing services (HTS) significantly declined from 12.6% in 2007 to 2.3% in 2015, 1.6% in 2016, and 1.5% in 2017 [6]. Known barriers to HIV testing uptake at facilities include perceptions of a lack of client confidentiality and fear of stigma and discrimination [3]. These barriers pose the greatest challenge to achieving the first “90” target in Vietnam.

Evidence suggests that community-based HIV testing can increase coverage and uptake of HIV testing, detect HIV infections earlier, and achieve a high HIV positivity yield when targeting KPs and the sexual partners (SP) of PLHIV. A systematic review and meta-analysis of community- and facility-based HIV testing in sub-Saharan Africa indicated that community-based HIV testing achieved higher HIV testing coverage and uptake and identified HIV-positive people with higher CD4 counts than facility-based HIV testing [7]. This meta-analysis also found that community-based HIV testing among the general population (GP) detected lower HIV positivity than facility-based HIV testing, whereas targeted community-based HIV testing for KPs and SP of PLHIV had the highest HIV positivity yield. Another systematic review and meta-analysis of 117 studies with a wide range of community-based HIV testing approaches (including door-to-door testing; mobile testing for GP, KPs, and adolescents; index testing; and self-testing) also found that community-based HIV testing achieved high rates of testing uptake, reached people with high CD4 counts, and linked people to care [8].

Task-shifting to lay providers has been recommended for a range of clinical care services, including HTS [9–12]. Use of lay providers offers the opportunity to deliver high-quality community-based HIV services and thus contribute to the 90-90-90 goals [12]. Evidence shows that HIV testing uptake increased after delegating HIV testing and counseling services to lay providers [13–15]. Studies conducted in Cambodia, Malawi, and South Africa comparing testing quality between lay providers and laboratory staff found that trained lay providers delivered high-quality, accurate HIV testing equivalent to that of health workers who had received more training [16–18]. Based on the evidence from these studies, the World Health Organization issued a strong recommendation for lay providers to perform HTS using HIV rapid diagnostic tests [19].

The low HIV testing coverage and low positivity yield of facility-based HTS imply that this approach is not enough to reach KPs in Vietnam. The question is: what HIV testing strategy would help reach those who are unreached among KPs and result in high positivity rates in Vietnam? In recognition of the potential of HIV lay provider testing, the Vietnam Ministry of Health (MOH) approved the implementation of a two-year pilot in October 2015 [20]. The United States Agency for International Development/PATH Healthy Markets project collaborated with the MOH and local partners to implement this pilot. An evaluation study was designed to assess the feasibility, acceptability, and effectiveness of lay provider HIV testing in Vietnam.

In this paper, we present findings from the evaluation of the lay provider HIV testing intervention pilot in Vietnam, with a focus on acceptability and effectiveness.

## Methods

### Study design and sample size

We conducted a cross-sectional survey to assess the acceptability of lay provider testing among KPs and SP of PLHIV, and an analysis of routine program data to evaluate the effectiveness of lay provider testing on HIV positivity yield and linkage to diagnosis and treatment.

Our hypothesis, based on results in other countries [13, 15], was that lay provider HIV testing would be effective at reaching high-risk populations that had previously never tested. We therefore defined lay testing acceptability as the proportion of first-time testers opting for lay provider testing. We estimated a sample size of 200 lay provider testing clients for each province and each type of test kit, and theorized that 50% of first-time testers would opt for lay provider HIV testing. With a finger-prick blood-based test used in all four provinces and an oral fluid-based test used in two provinces, the total sample was 1,230 for all four provinces.

### Description of the intervention

The lay provider HIV testing intervention was implemented in four provinces (Ho Chi Minh City, Hanoi, Nghe An, and Dien Bien), which were purposively selected based on the highest HIV burden, the largest number of KPs, and United States President’s Emergency Plan for AIDS Relief (PEPFAR) investments to reach the 90-90-90 targets in these locations. We implemented lay provider testing using a “test for triage” approach through KP-led community-based organizations (CBOs) and village health worker (VHW) networks where CBOs did not exist (see S1 Fig). The CBOs work with the Global Fund to Fight AIDS, Tuberculosis and Malaria and PEPFAR projects to reach KPs and link them to HIV services, while VHWs are local community volunteers who receive special training through the MOH to provide basic health care and counseling to people in their respective villages. CBO staff in urban areas and VHWs in rural mountainous areas received a three-and-a-half day training, including a two-day practicum on how to reach KPs and provide lay provider HIV testing. In sum, 143 staff of 16 CBOs and 322 VHWs in 11 rural mountainous districts participated in the training and provided HTS in this pilot.

As the goal of the lay provider HIV testing approach is to bring testing services closer to those who need them most (i.e., MSM and TGW, PWID, FSW, and SP of PLHIV), pilot HTS was provided in a flexible way to meet the needs of clients. The trained lay providers offered HTS at CBO offices, in clients’ homes, or at any private place preferred by the client, using a single rapid diagnostic test (RDT). Clients could receive this service outside working hours or on weekends. In rural mountainous areas, clients were offered only the finger-prick blood-based RDT (Alere Determine HIV-1/2 antibody); while in urban areas, clients were offered the choice of the finger-prick blood-based RDT (Alere Determine HIV-1/2 antibody) or an oral fluid-based RDT (OraSure Technologies’ OraQuick Rapid HIV-1/2 Antibody Test, after it became available in late August 2016).

Lay testers provided post-test counseling and referred clients who had an HIV-reactive test to the nearest health facility for HIV confirmatory testing (see S2 Fig). A venous blood sample was drawn at the health facility and transported to either the nearest MOH-certified district-level confirmatory laboratory (where three RDTs were used for HIV diagnosis), or to a provincial-level laboratory for enzyme immunoassay testing for confirmation of HIV diagnosis following the national HIV testing algorithm for strategy III describing the sequence of three different serological assays used for HIV diagnosis in Vietnam [21]. Clients received the confirmatory test result on the same day from the district confirmatory laboratory or within one week following testing at the provincial confirmatory laboratory. Clients who received a confirmed HIV-positive result were actively referred to a public or private clinic for initiation on ART. Clients with non-reactive test results received counseling to re-test after three months (if, potentially, they had been exposed to HIV within those three months) or six months, and were referred to a local health facility for HIV prevention services, such as opioid substitution therapy, pre-exposure prophylaxis (PrEP), non-occupational post-exposure prophylaxis, or sexually transmitted infection and/or hepatitis B and/or C virus management.

### Study population recruitment

Lay provider HIV testing was actively promoted by community outreach workers, and heavily advertised through Facebook and social networking applications (Grindr, Hornet), as well as during programs on MTV Vietnam or local television and radio, and on billboards in rural mountainous areas. People aged 18 and older who were HIV negative or of unknown status, and opted for HIV lay provider testing, were eligible for this cross-sectional survey. Prospective participants were provided with information about the study by trained researchers, who then assessed their eligibility against the study criteria and invited eligible participants to join the study. Oral informed consent was obtained from all study participants once the study’s purpose, process, and potential risks and benefits had been discussed.

A total of 1,230 study participants were enrolled in the cross-sectional survey. These study participants were selected from 13 of 16 CBOs (in Ho Chi Minh City and Hanoi) and 6 of 11 rural mountainous districts (in Nghe An and Dien Bien) implementing the lay provider HIV testing intervention.

### Data collection

The cross-sectional survey was conducted in two parts, before and after the client tested, using a structured questionnaire. The first half of the questionnaire included socio-demographic information, HIV risk behaviors, and HIV testing history; the second half covered satisfaction with the testing approach, willingness to pay, and preferences related to the type of HIV test, HIV tester, and location of HIV testing. Trained research staff conducted the interviews using computer-assisted personal interviewing or a paper-based questionnaire. In all, 1,230 individual interviews were completed in the period from January through December 2016.

We also reviewed and analyzed 54,837 records of clients who had received lay provider HTS in the 16 CBOs and 11 districts in the four provinces over nearly two years of the pilot (from December 2015 to September 2017). Lay providers collected and recorded data on service delivery for clients using a logbook. The logbook included information on each testing event and a unique code assigned to each client and linked to follow-up services for tracking confirmatory test and ART enrollment results. Lay providers actively assisted clients who tested HIV reactive to confirm HIV diagnosis and enroll on ART. All HIV-positive cases detected through lay provider testing were verified by district health centers or provincial HIV/AIDS centers and recorded in the MOH HIV.info system. District or CBO supervisors consolidated the monitoring data and reported the information to the Healthy Markets project on a monthly basis. Monitoring data were collected and used to analyze lay provider HIV testing, diagnosis, and treatment enrollment results over time.

### Data analysis

The primary outcomes of interest were the acceptability and effectiveness of lay provider HTS. In this study, we defined acceptability as the proportion of new HIV testers accessing lay provider HTS. We also described socio-demographic characteristics, client satisfaction, and the preferences of clients opting for HIV lay testing. We measured the effectiveness of lay provider HTS by assessing the HIV positivity rate and the successful referral rate to confirmatory testing and ART initiation.

The Kobo Toolbox application was employed for entry of data from the computer-assisted personal interviewing survey, and EpiData Version 3.1 was used for data entry from the paper-based questionnaires. All data were then converted to SPSS software for analysis. Data were analyzed using descriptive statistics and multivariate regression models in SPSS Version 22.0. Variables found to be statistically significant at a p value of less than 0.05 were included in the multivariate regression models. Multivariate regression models were used to examine the factors associated with having ever tested for HIV and preference for lay provider HIV testing. The results of the analysis are presented as adjusted odds ratios with 95% confidence intervals and interpreted as the odds of having ever tested for HIV and preference for lay provider HTS among those who were exposed or not exposed to the associated factor. Variables included in the multivariate regression models were age, education, income, occupation, ethnicity, marital status, residence, and KP group.

The HIV lay testing program monitoring data were analyzed to measure HIV positivity rates and successful rates of linkage to HIV confirmatory testing and ART enrollment. The cascade model applying the 2015 United States Agency for International Development LINKAGES (Linkages across the Continuum of HIV Services for Key Populations Affected by HIV) project guide was employed to visualize the results of this analysis [22].

### Ethical approval

The study was approved by the Institute of Social and Medical Studies’ Institutional Review Board in Hanoi, Vietnam, and PATH’s Research Determination Committee reviewed the protocol and determined it as non-research. Clients’ records for the monitoring data were anonymous and de-identified prior to analysis, as per the Vietnam MOH’s data protection and access policy.

## Results

Data were collected on 1,230 clients who opted for lay provider HIV testing services in four provinces, of whom 74% were KPs (MSM, PWID, and FSW) and the remaining were SP of PLHIV or GP. Nearly half (40.8%) were young people (aged 18 to 24 years), half (50.2%) reported income less than the mean (US$170 per month), more than half (57.6%) were single, and 65.8% had a high school education or less. Greater than half (56%) lived in an urban area (Ho Chi Minh City or Hanoi), 65% were of the Kinh majority ethnic group, and one-third (28.8%) were farmers (Table 1).

**Table 1:** Socio-demographic characteristics of the survey sample.

Characteristics	SP & GP n = 315 % (n)	MSM n = 553 % (n)	FSW n = 75 % (n)	PWID n = 287 % (n)	Total n = 1,230 % (n)
Age (in years)					
18 to 24	27.9 (88)	66.7 (369)	20.0 (15)	10.5 (30)	40.8 (502)
24 and older	72.1 (227)	33.3 (184)	80.0 (60)	89.5 (257)	59.2 (728)
Ethnic group					
Other minoritiesᵟ	61.6 (194)	4.2 (23)	42.7 (32)	64.5 (185)	35.3 (434)
Kinh majority	38.4 (121)	95.8 (530)	57.3 (43)	35.5 (102)	64.7 (796)
Education					
High school or lower	83.5 (263)	35.6 (197)	97.3 (73)	96.2 (276)	65.8 (809)
University or higher	16.5 (52)	64.4 (356)	2.7 (2)	3.8 (11)	34.2 (421)
Occupation					
Farmer	52.7 (166)	0.9 (5)	29.3 (22)	56.1 (161)	28.8 (354)
Small business/entertainment	8.9 (28)	30.4 (168)	53.3 (40)	4.2 (12)	20.2 (248)
Student	6.0 (19)	30.2 (167)	1.3 (1)	0 (0)	15.2 (187)
Freelance	26.0 (82)	22.4 (124)	13.3 (10)	32.1 (92)	25.0 (308)
Other	6.3 (20)	16.1 (89)	2.7 (2)	7.7 (22)	10.8 (133)
Income per month *(mean*: *VND3*,*832*,*965 or US$170)*					
Less than $170	69.5 (219)	31.3 (173)	53.3 (40)	64.8 (186)	50.2 (618)
$170 or more	30.5 (96)	68.7 (380)	46.7 (35)	35.2 (101)	49.8 (612)
Marital status					
Single	25.7 (81)	93.9 (519)	25.3 (19)	31.1 (89)	57.6 (708)
Currently married	63.8 (201)	2.5 (14)	30.7 (23)	57.3 (164)	32.7 (402)
Cohabitating with partner	2.9 (9)	2.7 (15)	12.0 (9)	1.7 (5)	3.1 (38)
Previously married	7.6 (24)	0.9 (5)	32.0 (24)	9.9 (28)	6.6 (81)
Residence					
Rural	88.5 (279)	0.9 (5)	49.3 (37)	76.3 (219)	43.9 (540)
Urban	11.5 (36)	99.1 (548)	50.7 (38)	23.7 (68)	56.1 (690)

ᵟOther minorities include Thai, H’Mong, Kho Mu, Hoa, and Khmer. n, sample size and subsample size. FSW, female sex workers; GP, general population; MSM, men who have sex with men; PWID, people who inject drugs; SP, sexual partners; VND, Vietnamese Dong.

### First-time and infrequent HIV testers

The majority (67%) of clients reported receiving their first HIV test through lay provider HTS, and nearly half (44.8%) of those who had ever been tested for HIV had not tested in the last 12 months (Table 2). There was a significantly higher proportion of first-time testers among SP & GP and PWID than among FSW and MSM (77.5% and 74.9% versus 60% and 57.9%, respectively) (p <0.001). There was also a significantly higher proportion of infrequent testers amid those who had ever been tested for HIV among FSW and PWID than among SP & GP and MSM (70% and 65.3% versus 45.1% and 35.2%, respectively) (p <0.001).

**Table 2:** Proportion of first-time testers and infrequent testers among lay provider HIV testing clients.

Behavior	SP & GP % (n)	MSM % (n)	FSW % (n)	PWID % (n)	Total % (n)	p value
Ever been HIV tested	n = 315	n = 553	n = 75	n = 287	n = 1,230	0.000***
No (first-time tester)	77.5 (244)	57.9 (320)	60.0 (45)	74.9 (215)	67.0 (824)	
Yes	22.5 (71)	42.1 (233)	40.0 (30)	25.1 (72)	33.0 (406)	
HIV tested in past 12 months	n = 71	n = 233	n = 30	n = 72	n = 406	0.000***
No (infrequent tester)	45.1 (32)	35.2 (82)	70.0 (21)	65.3 (47)	44.8 (182)	
Yes	54.9 (39)	64.8 (151)	30.0 (9)	34.7 (25)	55.2 (224)	

Chi-square test

*p <0.05

**p <0.01

***p <0.001.

n, sample size and subsample size. FSW, female sex workers; GP, general population; MSM, men who have sex with men; PWID, people who inject drugs; SP, sexual partners.

**Fig 1:**
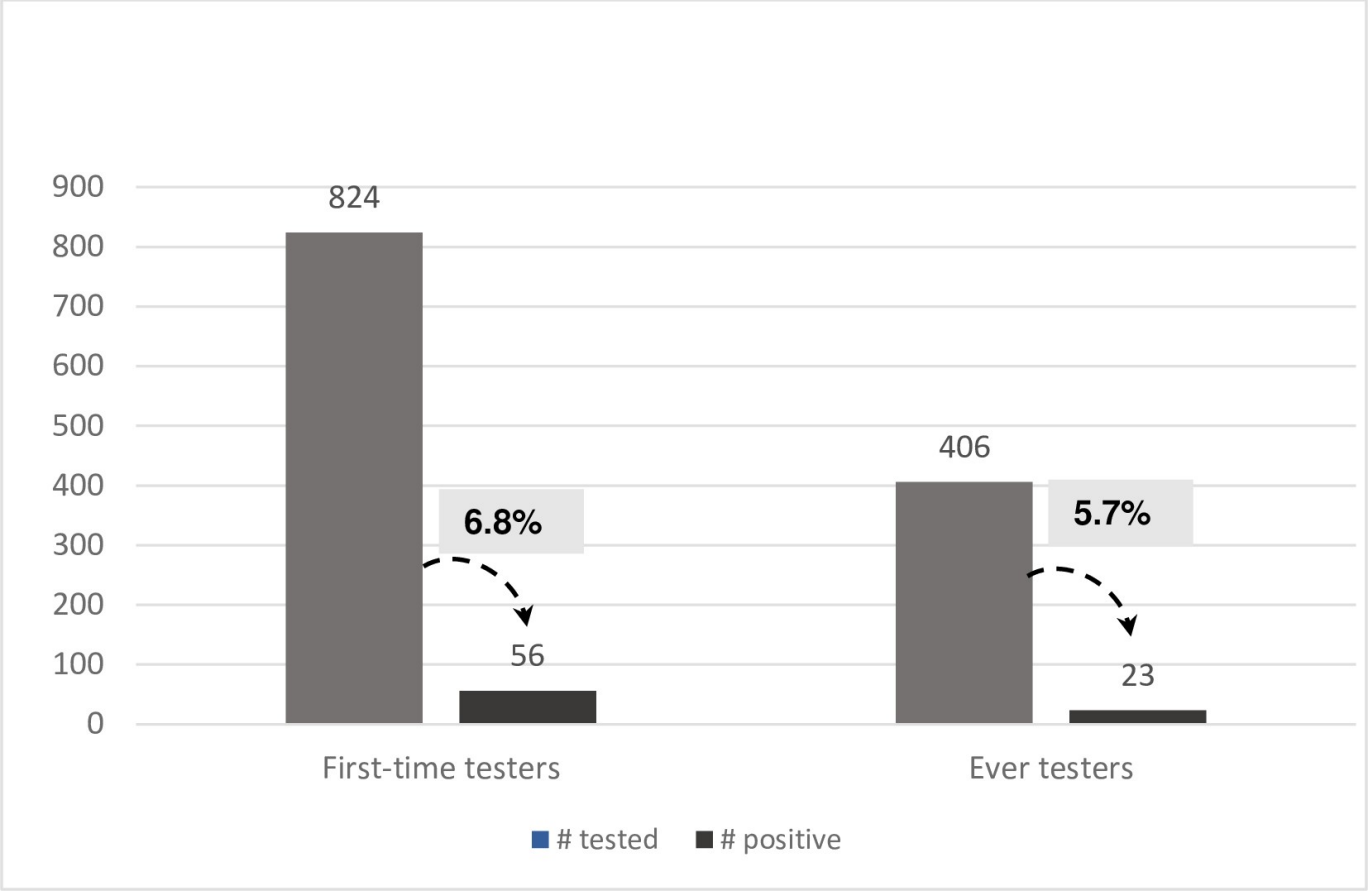
HIV positivity rates among first-time testers and ever testers.

HIV testing results among interviewed clients measured a higher HIV positivity rate among first-time testers than among ever testers (6.8% versus 5.7%) (Fig 1).

Univariate analysis comparison between having ever tested for HIV and first-time HIV testing revealed that MSM, FSW, urban residents, those with a university or higher education, those with a monthly income of $170 or higher, those with a small business or entertainment job, students, freelancers, and those of the Kinh majority ethnic group were more likely to have ever tested for HIV than SP & GP, rural residents, those with a high school or lower level of education, those with a monthly income of less than $170, and farmers (Table 3). Additionally, people currently married were less likely to have ever tested for HIV (i.e., first-time testers) than were single people. In the multivariate analysis, having ever tested for HIV and being an MSM (adjusted odds ratio [aOR] = 2.24; 95% confidence interval [CI]: 1.32–3.79) or being an FSW (aOR = 1.92; 95% CI: 1.05–3.50) or having a monthly income of $170 or more (aOR = 1.38; 95% CI: 1.03–1.85) remained significantly associated.

**Table 3:** Factors associated with ever HIV testing among lay provider HIV testing clients.

Factors	Univariate regression		Multivariate regression	
	Odds ratio (95% CI)	p value	Adjusted odds ratio (95% CI)	p value
Key population group				
SP & GP	Ref		Ref	
MSM	2.50 (1.82–3.42)	0.000***	2.24 (1.32–3.79)	0.003**
FSW	2.29 (1.34–3.90)	0.002**	1.92 (1.05–3.50)	0.033*
PWID	1.15 (0.79–1.67)	0.463	1.03 (0.69–1.53)	0.872
Residence				
Rural	Ref		Ref	
Urban	2.357 (1.8–3.0)	0.000	1.37 (0.78–2.40)	0.265
18 to 24	Ref		Ref	
24 and older	0.96 (0.75–1.22)	0.777	1.33 (0.96–1.83)	0.08
Education				
High school and lower	Ref		Ref	
University and higher	1.42 (1.11–1.82)	0.005**	1.10 (0.75–1.57)	0.65
Income (per month) *(mean*: *VND3*,*832*,*965 or US$170)*				
Less than mean	Ref		Ref	
Equal to or greater than mean	1.92 (1.51–2.45)	0.000***	1.38 (1.03–1.85)	0.033*
Occupation				
Farmer	Ref		Ref	
Small business/entertainment	2.43 (1.70–3.47)	0.000***	0.70 (0.38–1.33)	0.285
Student	1.79 (1.21–2.66)	0.004**	0.66 (0.33–1.35)	0.259
Freelance	2.29 (1.63–3.21)	0.000***	1.08 (0.64–1.81)	0.778
Other	1.69 (1.08–2.63)	0.020*	0.59 (0.31–1.14)	0.114
Ethnic group				
Other minoritiesᵟ	Ref		Ref	
Kinh majority	2.24 (1.71–2.93)	0.000***	1.43 (0.88–2.34)	0.142
Marital status				
Single	Ref		Ref	
Currently married	0.58 (0.44–0.76)	0.000***	1.28 (0.83–1.97)	0.254
Cohabitating with partner	1.40 (0.72–2.70)	0.312	1.60 (0.79–3.24)	0.185
Previously married	0.96 (0.59–1.56)	0.890	1.47 (0.82–2.63)	0.196

ᵟOther minorities include Thai, H’Mong, Kho Mu, Hoa, and Khmer. Logistic regression

*p <0.05

**p <0.01

***p <0.001.

CI, confidence interval; FSW, female sex workers; GP, general population; MSM, men who have sex with men; PWID, people who inject drugs; Ref, reference; SP, sexual partners; VND, Vietnamese Dong.

### Preference for lay provider HIV testing

In response to the question, if you opted for community-based HIV testing, whom would you most prefer to provide HIV testing for you, the majority (85.8%) preferred lay providers to health care providers (Table 4). A significantly higher proportion of MSM preferred lay provider HIV testing over health care provider testing, compared to PWID, FSW, and SP & GP (92.4% versus 78.7%, 80%, and 82.2%, respectively) (p <0.001). The majority (86.8%) also preferred community-based testing to facility-based testing. FSW and SP & GP were significantly more likely to prefer community-based testing than PWID and MSM (92% and 91.4% versus 83.3% and 85.3%, respectively) (p <0.01).

**Table 4:** Preference for lay provider HIV testing versus other HIV testing services.

Preference	SP & GP n = 315 % (n)	MSM n = 553 % (n)	FSW n = 75 % (n)	PWID n = 287 % (n)	Total n = 1,230 % (n)	p value
HIV testing performed by						0.000***
Lay provider	82.2 (259)	92.4 (511)	80.0 (60)	78.7 (226)	85.8 (1,056)	
Health care provider	14.6 (46)	1.6 (9)	14.7 (11)	18.2 (52)	9.6 (118)	
Other	3.2 (10)	6.0 (33)	5.3 (4)	3.1 (9)	4.6 (56)	
HIV testing place						0.005**
Community based	91.4 (288)	85.3 (472)	92.0 (69)	83.3 (239)	86.8 (1,068)	
Health facility based	4.2 (13)	2.9 (16)	1.3 (1)	4.9 (14)	3.6 (44)	
No preference/do not know	4.4 (14)	11.8 (65)	6.7 (5)	11.8 (34)	9.6 (118)	

Chi-square test

*p <0.05

**p <0.01

***p <0.001.

n, sample size and subsample size. FSW, female sex workers; GP, general population; MSM, men who have sex with men; PWID, people who inject drugs; SP, sexual partners.

Univariate analysis comparison between preference and non-preference for lay provider HTS found that MSM, urban residents, those with a university or higher education, students, and those of the Kinh ethnicity were more likely to prefer lay provider HTS than SP & GP, rural residents, those with a high school or lower education, farmers, and ethnic minorities (Table 5). In contrast, people older than 24 years, currently married, or previously married were less likely to prefer lay provider HTS than people aged 18 to 24 years and single. The multivariate analysis confirmed a significant association between lay provider HTS preference and urban residence (aOR = 2.50; 95% CI: 1.30–4.90) and a university or higher education (aOR = 1.83; 95% CI: 1.05–3.20). The multivariate analysis also confirmed a significant inverse association of lay provider HTS preference and occupation, including those working in small businesses and entertainment (aOR = 0.23; 95% CI: 0.10–0.46) and freelancers (aOR = 0.57; 95% CI: 0.33–0.98). In other words, freelancers and those working in small businesses and entertainment services were far less likely to prefer lay provider HTS.

**Table 5:** Factors associated with preference for lay provider HIV testing.

Factors	Univariate regression		Multivariate regression	
	Odds ratio (95% CI)	p value	Adjusted odds ratio (95% CI)	p value
Key population group				
SP & GP	Ref		Ref	
MSM	2.63 (1.71–4.03)	0.000***	1.58 (0.79–3.2)	0.197
FSW	0.86 (0.45–1.63)	0.654	1.12 (0.54–2.33)	0.760
PWID	0.80 (0.53–1.20)	0.282	0.68 (0.44–1.05)	0.080
Residence				
Rural	Ref		Ref	
Urban	2.50 (1.79–3.48)	0.000***	2.50 (1.30–4.90)	0.006**
Age (years)				
18 to 24	Ref		Ref	
Older than 24	0.64 (0.46–0.91)	0.013*	1.28 (0.83–1.99)	0.258
Education				
High school or lower	Ref		Ref	
University or higher	2.48 (1.66–3.69)	0.000***	1.83 (1.05–3.20)	0.034*
Income (per month) *(mean*: *VND3*,*832*,*965* or *US$170)*				
Less than mean	Ref		Ref	
Equal to or greater than mean	1.16 (0.84–1.60)	0.362	0.79 (0.54–1.19)	0.265
Occupation				
Farmer	Ref		Ref	
Small business/entertainment	1.11 (0.71–1.73)	0.625	0.23 (0.10–0.46)	0.000***
Student	3.03 (1.59–5.79)	0.001**	0.40 (0.15–1.06)	0.066
Freelance	1.24 (0.81–1.90)	0.301	0.57 (0.33–0.98)	0.044*
Other	1.33 (0.75–2.34)	0.325	0.34 (0.16–0.72)	0.005
Ethnic group				
Other minoritiesᵟ	Ref		Ref	
Kinh majority	1.88 (1.36–2.60)	0.000***	1.28 (0.74–2.2)	0.376
Marital status				
Single	Ref		Ref	
Currently married	0.50 (0.35–0.70)	0.000***	0.89 (0.55–1.46)	0.659
Cohabitating with partner	0.78 (0.29–2.06)	0.619	0.99 (0.35–2.76)	0.993
Previously married	0.44 (0.24–0.80)	0.007**	0.73 (0.37–1.460)	0.376

ᵟOther minorities include Thai, H’Mong, Kho Mu, Hoa, and Khmer. Logistic regression

*p <0.05

**p <0.01

***p <0.001.

CI, confidence interval; FSW, female sex workers; GP, general population; MSM, men who have sex with men; PWID, people who inject drugs; Ref, reference; SP, sexual partners; VND, Vietnamese Dong.

### Satisfaction with lay provider HIV testing

Overall, the vast majority (93%) of clients reported being satisfied or very satisfied with their lay provider HTS experience (Table 6). A significantly higher proportion of MSM and FSW compared to PWID and SP & GP were satisfied or very satisfied with their lay provider HTS experience (98.2% and 93.4% versus 86.7% and 89.9%, respectively) (p <0.001). Satisfaction was also reflected in 95.4% of clients reporting that they would recommend lay provider HTS to their friends and family.

**Table 6:** Client satisfaction with lay provider HIV testing.

Value	SP & GP n = 315 % (n)	MSM n = 553 % (n)	FSW n = 75 % (n)	PWID n = 287 % (n)	Total n = 1,230 % (n)	p value
Satisfaction with lay provider HIV testing						0.000***
Very satisfied	30.2 (95)	27.7 (153)	42.7 (32)	39.0 (112)	31.9 (392)	
Satisfied	59.7 (188)	70.5 (390)	50.7 (38)	47.7 (137)	61.2 (753)	
Neutral	10.2 (32)	1.8 (10)	6.7 (5)	13.2 (38)	6.9 (85)	
Would recommend lay provider HIV testing to other people (friends and family)						0.092
Yes	96.2 (303)	96.0 (531)	94.7 (71)	93.4 (268)	95.4 (1,173)	
No	2.5 (8)	3.3 (18)	5.3 (4)	3.5 (10)	3.3 (40)	
Do not know/unsure	1.3 (4)	0.7 (4)	0 (0)	3.1 (9)	1.4 (17)	

Chi-square test

*p <0.05

**p <0.01

***p <0.001.

n, sample size and subsample size. FSW, female sex workers; GP, general population; MSM, men who have sex with men; PWID, people who inject drugs; SP, sexual partners.

### Reasons for opting for lay provider HIV testing

**Fig 2:**
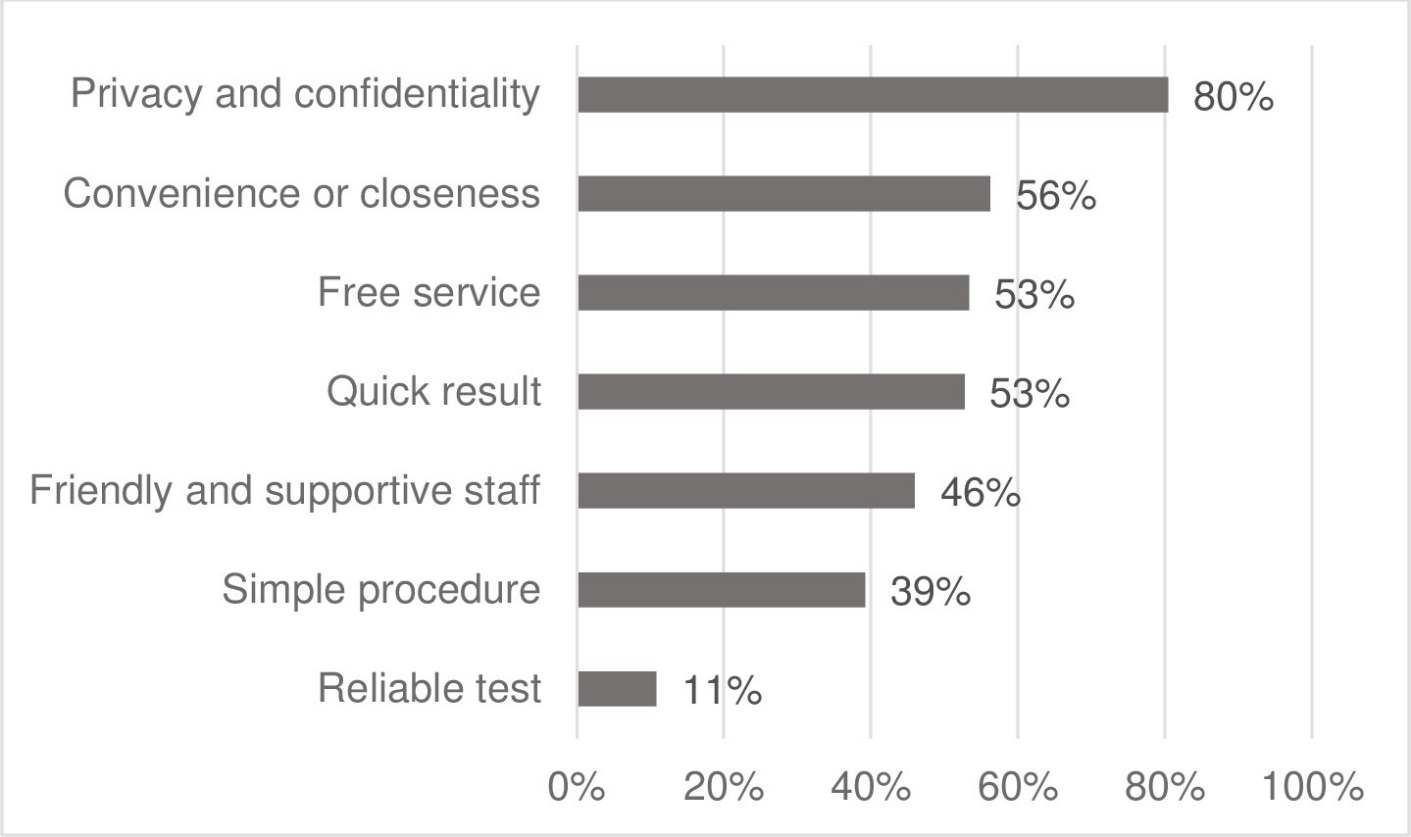
Reasons for opting for lay provider HIV testing.

In our study, the top five reasons that respondents opted for lay provider HTS were privacy and confidentiality (80%), convenience or closeness (56%), free service (53%), quick results (53%), and friendly and supportive staff (46%) (Fig 2).

### HIV positivity rate

Overall, the HIV positivity rate among populations opting for lay provider HIV testing was 4.1%. Further, the mean number of clients who needed to be tested by lay providers in order to identify a person with HIV was 24 (Table 7). The HIV positivity rates were higher in MSM, FSW, and PWID than in SP and GP (5.9%, 4.8%, and 3.8% versus 1.7% and 0.9%, respectively).

**Table 7:** Number of clients needed to screen to identify a person with HIV in lay provider HIV testing services.

Population	Number tested	Number positive	Positivity rate	Number needed to screen
MSM	23,514	1,391	5.9%	17
FSW	1,817	88	4.8%	21
PWID	13,624	525	3.8%	26
SP	11,297	189	1.7%	60
GP	4,585	41	0.9%	112
**Total**	**54,837**	**2,234**	**4.1%**	**24**

FSW, female sex workers; GP, general population; MSM, men who have sex with men; PWID, people who inject drugs; SP, sexual partners.

### Linkage to care

**Fig 3:**
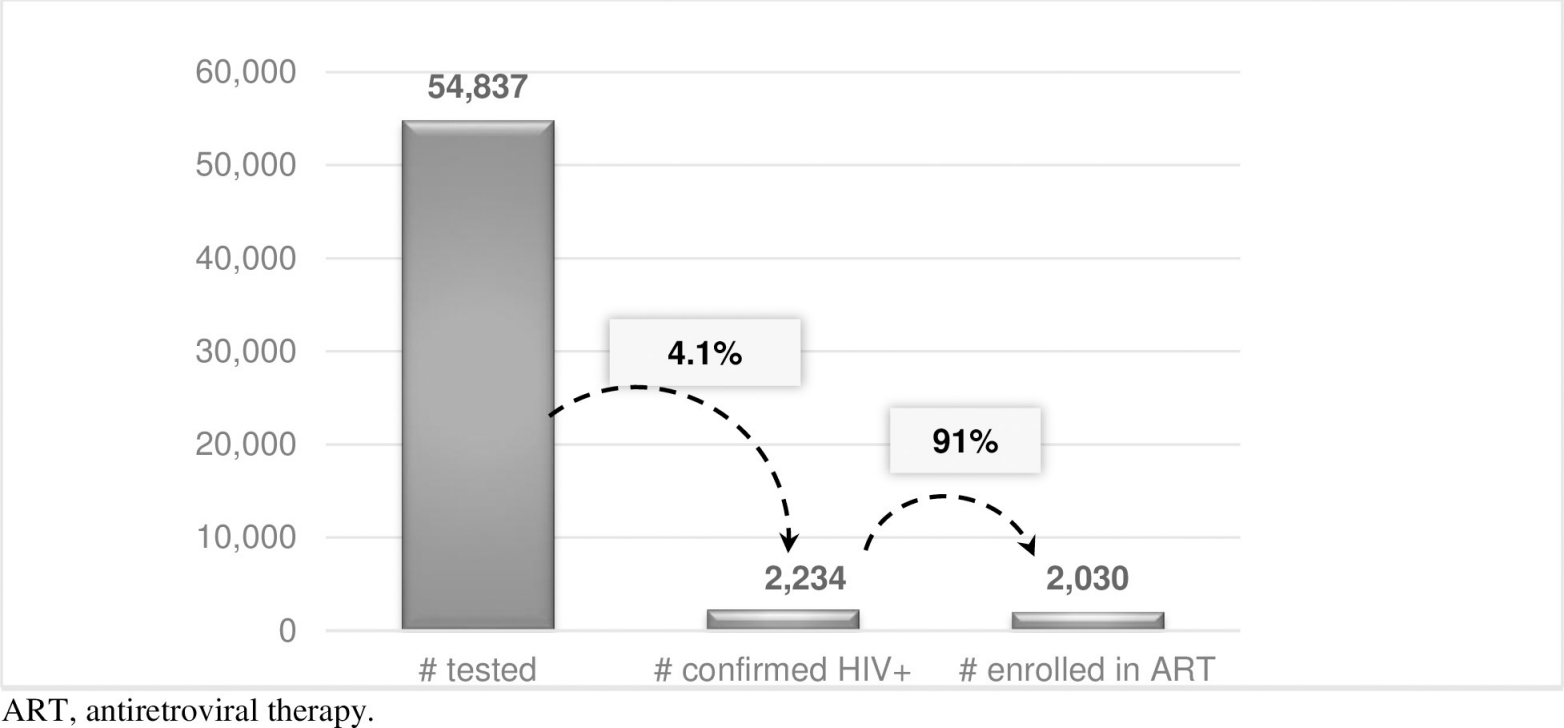
Linkage to care with lay provider HIV testing services.

Overall, 91% of clients who were diagnosed HIV positive through lay provider services were enrolled on ART (follow-up up to three months after being diagnosed to report to the project on a quarterly basis) (Fig 3). The proportion of populations that were linked to ART services was higher among PWID, MSM, and SP than among FSW and GP (94%, 93%, and 91% versus 66% and 37%, respectively).

## Discussion

Our findings demonstrate that lay provider HIV testing following the test for triage approach was successful in reaching those who had not previously tested for HIV, and resulted in a high HIV positivity yield and ART initiation rate, thereby supporting efforts to accelerate Vietnam’s attainment of the 90-90-90 targets. The high proportion of lay provider HTS clients who were first-time HIV testers (67%) and the higher HIV positivity rate among the first-time testers compared to ever HIV testers suggest that lay provider HIV testing is an effective approach for reaching people not previously reached by facility-based testing, and who are at greater risk for HIV. Previous studies conducted in generalized epidemic settings found a varied proportion of first-time testers reached with community-based HIV testing, ranging from 9% to 79% [8]. We theorize that the high proportion of first-time testers opting for lay provider HTS may decline over time, as more and more repeat testers make use of this preferred service.

Within the context of low HIV testing uptake, a new HIV testing approach that is highly acceptable to KPs could be “game changing” in boosting HIV testing uptake. Very high satisfaction with and preference for lay provider HTS imply that this approach was highly acceptable to clients, particularly KPs. This finding is consistent with previous studies reporting satisfaction with lay provider HTS [19]. Our findings suggest that the lay provider HIV testing approach addressed barriers to HIV testing that previously inhibited KP uptake of existing facility-based HTS, such as perceived lack of confidentiality, fear of stigma and discrimination, inconvenient service opening times and distance, and long waiting times for test results.

Our lay provider HIV testing intervention reached a large number of clients (54,837) with an overall HIV positivity rate of 4.1%—nearly three times higher than the facility-based testing HIV-positive rate in the country during the same period (estimated 1.6% in 2016 and 1.5% in 2017) [6]. Interestingly, community-based HTS offered by health care providers through door-to-door testing, mobile testing, workplace testing, and school-based testing, resulted in a lower HIV positivity rate than for facility-based HTS [8].

In our study, the HIV positivity rate was highest among key populations (MSM, FSW, and PWID), followed by SP of PLHIV, and then GP. These results mirror Vietnam’s concentrated HIV epidemic, where there is a higher HIV prevalence among KPs than GP.

We believe that one of the key factors to the success of lay provider HIV testing is that it is offered by people from the community who are trusted by HIV-affected populations. It is particularly true with HIV testing delivered by MSM-led CBOs, which we reported in a recent paper [23]. This is not unique to Vietnam; studies evaluating similar approaches in Thailand have concluded that KP-led health services were of high quality and trusted by the community, resulting in greater HIV testing uptake and ART enrollment [24, 25].

Since Vietnam adopted the “test and treat” strategy in 2015 to accelerate progress toward achieving the 90-90-90 targets, all people diagnosed with HIV are now eligible for enrollment on ART without having CD4 counts. A very high proportion (91%) of HIV-positive clients diagnosed through lay provider HTS successfully initiated ART enrollment. The success of linkage to care was likely due to lay providers in this pilot not only delivering HTS, but also offering clients ongoing supportive linkage and follow-up with health facilities for confirmatory testing and ART enrollment. This finding is consistent with previous studies in sub-Saharan Africa, which indicated that community testing with facilitated linkage (e.g., counselor follow-up to support linkage) achieved high linkage to care (87% to 98%) [7]. In low ART enrollment contexts like Vietnam (estimated 47% ART coverage in 2016), the lay provider HIV testing approach could play a major role in efforts to achieve the second objective of the 90-90-90 targets (90% of PLHIV on ART).

There were three key limitations to this study. First, the study included only clients who opted for lay provider HIV testing; due to limited resources, we did not include a control group of clients who opted for other HTS (e.g., facility-based HTS). This restricted our ability to determine the true impact of the intervention on key outcomes. However, this was not within the scope of our study, which using operations research aimed to assess lay provider HTS acceptability and effectiveness in a real-world setting. Resources permitting, a follow-up controlled study would be ideal to examine the impact of the intervention in a concentrated epidemic setting. Second, routine monitoring data from logbooks were used in the analysis of the intervention’s effectiveness, reported in accordance with PEPFAR’s Monitoring, Evaluation, and Reporting indicators, which prevented us from conducting statistical power analysis. Third, data on CD4 counts or viral load tests among clients diagnosed as the result of the intervention were not available or difficult to track in a number of health facilities. Thus, we were not able to measure the effectiveness of lay provider HIV testing in detecting PLHIV earlier in the course of HIV infection.

Despite these limitations, our study had unique strengths. It provided empirical evidence on the effectiveness of lay provider HIV testing—a groundbreaking approach for Vietnam: for the first time ever, non-health care workers representing KPs and frontline village health volunteers demonstrated capacity to provide highly effective HIV testing services. Based on the results of the pilot, the Vietnam MOH developed and approved the national guidelines on community-based HIV testing and counseling in April 2018. These guidelines provide the foundation for further scale-up of lay provider HIV testing [26]. The pilot also spurred wider adoption of lay provider HIV testing, which was included in the 2018–2020 Global Fund concept note and in PEPFAR programming. As a result, lay provider HIV testing services are now available in 32 of 63 provinces of the country.

## Conclusions

Our findings suggest that lay provider HIV testing is an effective approach to reach those at risk of HIV who have never tested or test infrequently. Lay provider HIV testing can be a critical addition to efforts to achieve the 90-90-90 targets and to reaching the “last mile” in Vietnam.

## Supporting information

S1 Fig “Test for triage” approach to lay provider HIV testing. (TIF)

S2 Fig Protocol for lay provider HIV testing. (TIF)

S1 Text Questionnaire translated into English. (PDF)

S2 Text Questionnaire in original Vietnamese. (PDF)

S1 Dataset Dataset. (XLSX)
