# Supplementary figures and images for: Lay provider HIV testing: A promising strategy to reach the undiagnosed key populations in Vietnam
Source: PLoS One. 2018 Dec 31;13(12):e0210063. doi: 10.1371/journal.pone.0210063 (PMC6312239; doi:10.1371/journal.pone.0210063)

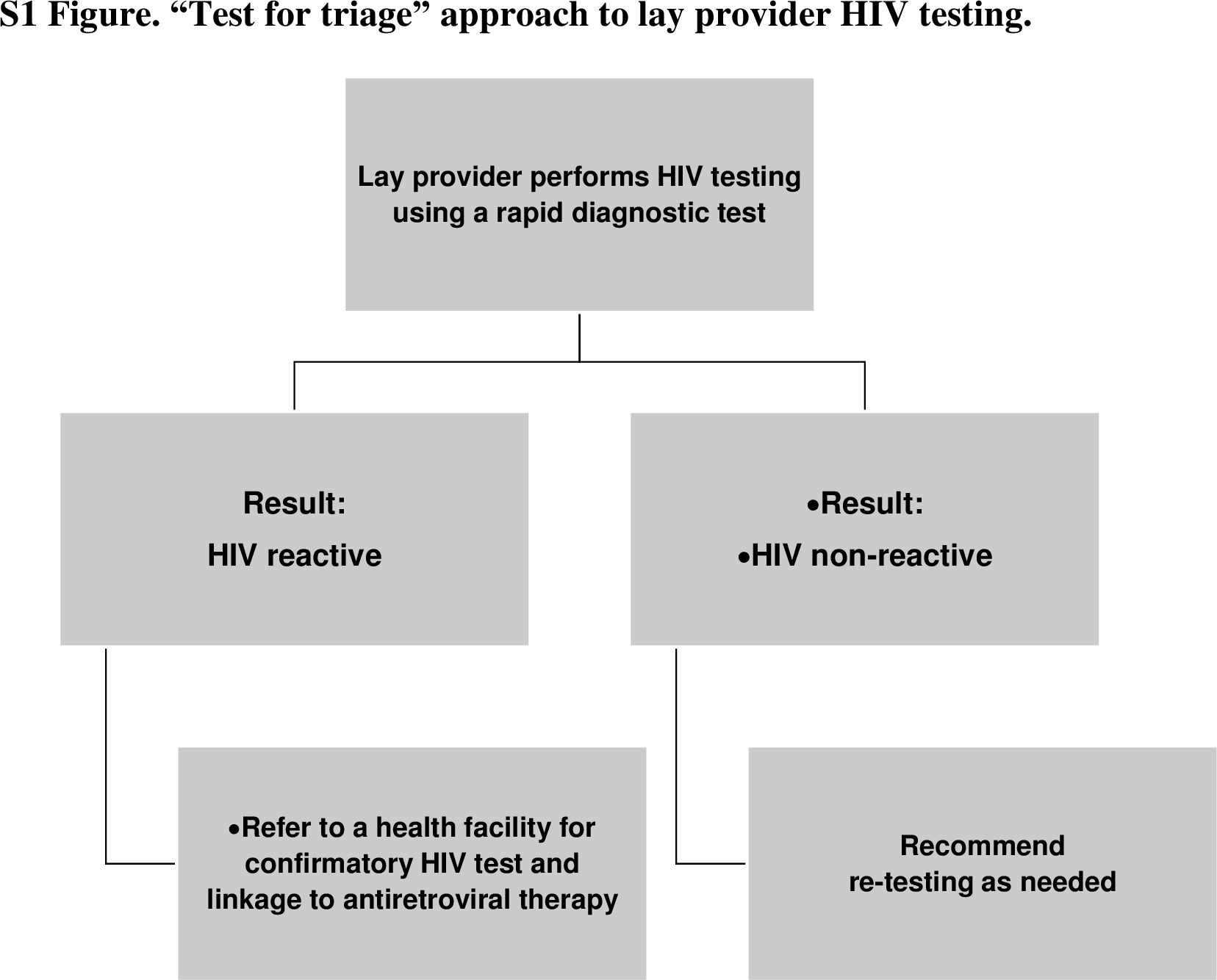

Supplement: S1 Fig — (TIF) [file pone.0210063.s001.tif]

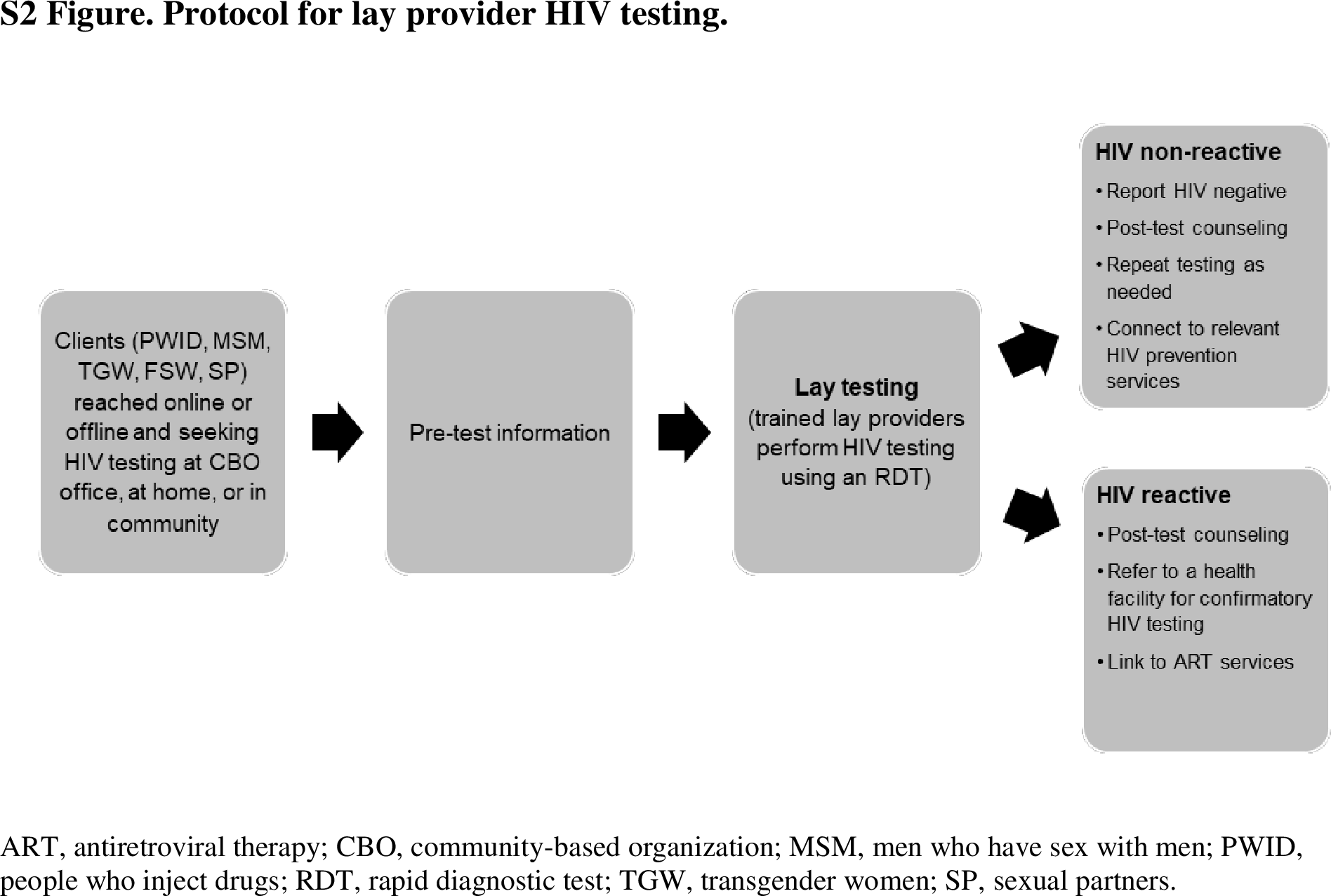

Supplement: S2 Fig — (TIF) [file pone.0210063.s002.tif]
