# Supplementary material for: Lay provider HIV testing: A promising strategy to reach the undiagnosed key populations in Vietnam
Source: PLoS One. 2018 Dec 31;13(12):e0210063. doi: 10.1371/journal.pone.0210063 (PMC6312239; doi:10.1371/journal.pone.0210063)
Supplement: S1 Text — (PDF) [file pone.0210063.s003.pdf]

QUESTIONNAIRES ON HIV LAY TESTING (TRANSLATED)

1. Date of performing a test (DD/MM/YY): ☐☐ / ☐☐ / ☐☐☐☐
2. Lay tester ID: ☐☐☐☐☐☐
3. Client ID: ☐☐☐☐☐☐☐☐☐☐
4. City/Province:
   1. Dien Bien 2. Nghe An 3. Ha Noi 4. Ho Chi Minh
5. District:……………………………….…
6. Observer ID: ……………………………

PART A: SCREENING

No	Question	Answer	Code	Note
A1	Have you ever been tested for HIV?	Yes	1	
		No	0	0 → B1
A2	Have you tested for HIV in the last 12 months?	Yes	1	
		No	0	
A3	Where was your most recent HIV test?	HTC site	1	
		Public hospital/clinic	2	
		Private hospital/clinic	3	
		Rehab center (05 – 06)	4	
		Test performed by village health worker/ community staff/ CBO	5	Thank you and **end the interview**
		Self-testing	6	
		Mobile testing	7	
		Other (please specify) ______________	88	
		Don't remember/ don't know	99	
A4	Can you share with me the result of your most recent HIV test?<br>*You can refuse to answer this question if you don't want to*	Positive	1	Thank you and **end the interview,** introduce to counselor for referral to C&T
		Negative	2	

		Unidentified	3	
		Don't know/ don't answer	99	

PART B: DEMOGRAPHIC INFORMATION

No	Question	Answer	Code	Note
B1	What is your self-identified sex?	Male	1	
		Female	2	
		Male to Female transgender	3	
		Female to Male transgender	4	
B2	Which year were you born? *(Calendar year)*	Year	________	
B3	What is your ethnicity?	Kinh	1	
		Thai	2	
		KhoMu	3	
		H'Mong	4	
		Dao	5	
		Hoa	6	
		Khmer	7	
		Other (please specify) ______________	88	
B4	What is your **highest level of education**?	Illiterate	1	
		Primary school (grades 1-5)	2	
		Secondary school (grades 6-9)	3	
		High School (grades 10-12)	4	
		Junior college, college, university, post-graduation	5	
B5	What is your main job? *(The job that takes most of time and generates income)*	Farming, fishery, forestry	1	
		Worker in factory/construction	2	
		White color employee in government sector/private sector	3	
		Entertainment/tourism staff	4	
		Selling things, doing small business, services	5	

No	Question	Answer	Code	Note
		Student	6	
		Sex Work	7	
		Freelance	8	
		Unemployed	9	
		Other (please specify) ___________	88	
B6	What was your average income per month in the last year?<br><br>*(including all sources of income)*	Amount _______________		
		No answer	99	
B7	What is your current marital status?	Single	1	
		Married	2	
		Living with partner but not married	3	
		Seprated/divorced/widowed	4	
		Other (please specify) ___________	88	
B8	Have you ever had sex in exchange for money or other assets?	Yes	1	
		Never	0	
		Do not answer	99	
B9	Have you ever had anal sex with other men?	N/A (in case of FEMALE or FEMALE to MALE TRANSGENDER)	2	
		Yes	1	Only ask male or male to female transgender
		Never	0	
		Do not answer	99	
B10	Have you ever injected a substance (drug)?	Yes	1	
		Never	0	
		Do not answer	99	

PART C: PRE-TEST

No	Question	Answer	Code	Note
C1	Have you ever heard of a HIV test performed by a non-health care worker such as a: village health worker, community staff, or CBO workers?	Yes	1	
		No	0	0 → C3
		Don't remember	99	99 → C3
C2	If yes, where did you get that piece of information? ***(Multiple choices)***	Community outreach workers	1	
		Health staff	2	
		Friends	3	
		Relatives	4	
		Spouse/partners	5	
		Facebook	6	
		Internet	7	
		TV, radio	8	
		Leaflets	9	
		Billboards	10	
		Meetings, communication sessions in community	11	
		Other (please specify)	88	
C3	Why do you choose the test performed by a non-health care worker (village health worker/ community staff/ CBO)? ***(Multiple choices)***	anonymous	1	
		Ensuring confidentiality	2	
		Ensuring privacy	3	
		Simple procedures	4	
		Near and convenient for travelling	5	
		Flexible time	6	
		Knowing result quickly	7	

No	Question	Answer	Code	Note
		Reasonable price	8	
		Free	9	
		Receiving clear and detailed counseling	10	
		Friendly and considerate staff	11	
		Accurate test	12	
		Receiving instruction and support in referral to health facilities	13	
		Introduced by community outreach workers	14	
		Introduced by health staff	15	
		Other (please specify)	88	
C4	To help you decide to take this test, what information do you need the most?<br><br>***(Choose the 3 most important answers)***<br><br>***Show the answers.***	Price of the test	1	
		Accuracy of the test	2	
		Confidentiality of the test	3	
		Duration of the test and getting result	4	
		Process of performing the test	5	
		The place to take the test, how it looks like	6	
		Testing equipment/instruments	7	
		Who is the tester amdtheir capacity	8	
		Attitudes of the staff/testers	9	
		Quality of counseling/ persuading skills of counselor	10	
		Connection to other services such as confirmative test, C&T	11	
		Other (please specify) ________	88	
C5	Who/where do you want to receive these pieces of	Community outreach worker	1	
		Health staff	2	

No	Question	Answer	Code	Note
	information?	Friends	3	
		Relatives	4	
		Spouse/partners	5	
		Facebook	6	
		Internet	7	
		TV, radio	8	
		Leaflets	9	
		Billboards	10	
		Meetings, communication sessions in community meetings	11	
		Other (please specify)	88	

AFTER PART C, LAY TESTER STARTS PRE-TEST COUNSELING AND PERFORM THE TEST

(Mark X in all appropriate columns)

PART D1. PRE-TEST OBSERVATION

#	Activities	Yes	No	Meet the standard	Notes
	**PREPARE MATERIALS**				
D1	Test card Determine HIV 1/2			NA	
D2	Lancet			NA	
D3	Chase buffer			NA	
D4	Capillary tube			NA	
D5	Sterile gauze			NA	
D6	Antiseptive wipes			NA	
D7	Bandage			NA	
D8	Disposable gloves			NA	
D9	Tray			NA	
D10	3 seperate waste bags			NA	
D11	Watch/clock (any type)			NA	
	**PRE-TEST COUNSELING, EMPHASIZE:**				
D12	Taking a test is voluntary			NA	
D13	Related information is kept confidential			NA	

#	Activities	Yes	No	Meet the standard	Notes
	and privacy is ensured				
D14	Explain process of testing (prick finger, collect the blood with capillary tube, transfer the blood to the sample pad, wait and read the result)			NA	
D15	Interprete the result : *This test is accurate, but it is only a screening test for triage, not confirmative test.*			NA	
D16	Confirm if the client agrees to take the test (**Meet the standard:** Obtain written or verbal consent consent)				

PART D2. OBSERVATION OF PERFORMING A TEST

#	Activities	Yes	No	Meet the standard	Notes
**TAKING THE TEST**					
D17	Write down client code on the sample pad (**Meet the standard:** the client code written on the sample strip is <u>the same</u> with the client code in the logbook)				Client code has 4 digits
D18	Lay tester washing hands (with water or hand washing soap provided by the project)			NA	
D19	Lay tester wearing gloves			NA	
D20	Tear one strip and remove the cover of the test pad (**Meet the standard:** without touching finger(s) on the sample pad)				
D21	Massage the finger (*middle or ring fingers*)			NA	

#	Activities	Yes	No	Meet the standard	Notes
D22	Clean the finger of the client with an antiseptic wipe (middle finger or ring-finger)			NA	
D23	Performing finger prick<br>(Circle the appropriate option)	1. One time<br>2. Two times<br>3. Three times<br>4. Three times but fail			**If circle 4. -> end observation**
D24	Wipe away the first drop of blood with a sterile gauze pad			NA	
D25	Collect the blood with a capillary tube<br>(**Meet the standard:** fill the tube with blood between the 2 marked lines without too many air bubbles)				
D26	Bandage to stop the bleeding			NA	
D27	Transfer the blood to the sample pad (**Meet the standard:** ensure the sample is fully dispensed on the sample pad)				
D28	Apply one drop of chase buffer to the sample pad<br>(**Meet the standard**: The tip of the chase buffer does not touch the sample on the test strip)				
D29	Read the results timely?<br>(**Meet the standard**: Reading the results between 15-60 minutes, not reading the results after 60 mins)				
D30	Read the results correctly?<br>(**Meet the standard**:				Observer checks if lay

#	Activities	Yes	No	Meet the standard	Notes
	- *Non-reactive: 1 red bar appears in control window*<br>- *Reactive: 2 red bars appear in control window and patient window*<br>- *Invalid: there is no red bar in the control window of the strip or a red bar appears in the patient window)*				tester reads result correctly or not
D31	Results of the test read by the observer	1. Non-reactive<br>2. Reactive<br>3. Invalid			Observer circles the appropriate option

PART D3. POST-TEST OBSERVATION

#	Activities	Yes	No	Meet the standard	Notes
**POST-TEST COUNSELING**					
D32	Reconfirm confidentiality of information related to the test and the client			NA	
**NON-REACTIVE RESULT**					
D33	Explain the non-reactive result:<br>(**Meet the standard**: *the result is non-reactive. This result confirms 3 months ago you were not infected with HIV. But, this result does not reflect your status within the past 3 months)*				
D34	Talk about HIV prevention measures (e.g. condom use, sterile needles & syringes, methadone maintenance treatment)			NA	

D35	Make an arrangement for retaking the test (**Meet the standard:** retake a test after 3 months, if the client had exposed to risk behavior in the last 3 months or take a test periodically every 6 months for any KP)				
**REACTIVE RESULT**					
D36	Explain the reactive result: (**Meet the standard**: *the result is reactive, which means you are likely to be infected with HIV. However, this is just a screening test, not confirmative. It is essential for you to take another test in health facility to confirm your status as early as possible).*				
D37	Talk about the necessity of taking a confirmative test			NA	
D38	Introduce client to a health facility to have confirmative test			NA	
D39	Show empathy and willingness to support for the client to have a confirmative test (**Meet the standard**: Write and give a referral slip to the client, and make an appointment with the health facility for the client, and offer accompanying him/her to the health facility, if he/she agrees)				
**INVALID RESULT**					
D40	Admit mistake and apologize client for inconvenience of retaking the test			NA	
D41	Ask for permission to retake the test			NA	

D42	Encourage client to take their partners to take a test			NA	
**FINISH UP**					
D43	Collect rubbish and medical waste for disposal according to regulations<br>(**Meet the standard:** contain in 3 separate waste bags/ dustbins: 1. Sample pad; 2. Lancet; 3. Rubbish)				
D44	Wash hands			NA	
D45	Note down client's information in the logbook			NA	

Note: Mark X in both "**Yes**" and "**Meet standard**" columns, if relevant in rows with "**Meet standard:…**"

PART E: POST-TEST PROCEDURE (INTERVIEW CLIENT INDIVIDUALLY)

#	Question	Answer	Code	Notes
E1	In general, are you satisfied with the testing service you have received?	Very satisfied	1	
		Satisfied	2	
		Neutral	3	**3** → E4
		Unsatisfied	4	**4** → E3
		Very unsatisfied	5	**5** → E3
E2	If yes, what are you satisfied with?<br>**(Multiple choices)**	Appropriate place for testing (convenient/ clean/ private)	1	After E2 → move to E4
		Flexible time, short waiting time, quick result	2	

#	Question	Answer	Code	Notes
		Friendly and considerate staff	3	
		Staff has good skills of performing a test	4	
		Simple and painless sample taking (finger prick, oral swab)	5	
		Easy to understand and detailed counseling	6	
		Ensuring confidentiality of the test	7	
		Free	8	
		Reasonable price	9	
		Receiving instruction and referral to other services such as confirmative test, C&T	10	
		Other (Please specify) ________________	88	
E3	If NO, what are you unsatisfied with? ***(Multiple choices)***	Inapropriate place for testing (far/unclean/ not private)	1	
		Inappropriate schedule for the test, long waiting time	2	
		Staffhas inapropriate attitude (unfriendly)	3	
		skills of the staff to take the test are not good	4	
		Counseling is done in a unthorough and hasty manner	5	

#	Question	Answer	Code	Notes
		Confidentiality and privacy are not ensured	6	
		The test is costly	7	
		No referral to other services such as confirmative test, C&T	8	
		Unclear testing result	9	
		Other (please specify)	88	
E4	**Where** do you want a village health staff/ community staff/ CBO to perform HIV lay testing **the most**?<br><br>*Show or read out loud the answers*<br><br>***Choose one answer***	At your house	1	
		At your workplace	2	
		At lay tester's house	3	
		At lay tester's workplace (e.g. CBO office)	4	
		At commune health station	5	
		In a private place, not the above-mentioned places	6	
		Wherever, I don't care	7	
		Other (please specify) ____________	88	
E5	If you continue taking an HIV test in the community, who do you want **the most** to perform the test for you?	Community staff/ CBO	1	
		Village health worker	2	
		Commune health station staff	3	
		District health staff doing HIV mobile testing	4	

#	Question	Answer	Code	Notes
	Show or read out loud the answers<br>***Choose one answer***	Self-test	5	
		Other (please specify)	88	
		Won't do it again/ Don't know/ Don't answer	99	
E6	If you continue taking an HIV test, what **form of sample taking** do you **like the most?**<br>(explain the difference between the three options and show a picture)<br>*Show or read out loud the answers*<br>***Choose one answer***	Oral swab	1	
		Finger prick	2	
		Venipuncture	3	
		Any form is OK	4	
		Won't do it again/ Don't know/ Don't answer	99	
E7	If you had the option for a HIV self-test (explain what it means), would you rather test yourself instead of being tested by someone else?	Yes	1	
		No	2	
		No preference	3	
		Don't know/don't answer	99	
E8	Now that you have tested for HIV in the community, can you tell us if you prefer:<br>Show *or read out loud the answers*<br>(choose one answer)	Testing in the community as you did today	1	
		Testing in a fixed HIV testing site such as a VCT center of other public HIV testing site	2	
		Testing in a private clinic or hospital	3	

#	Question	Answer	Code	Notes
		No preference	4	
		Don't know/don't answer	99	
E9	Do you want to **introduce** the testing service that you have today to other people (for example your spouse/partner, friends or family members?	Yes	1	
		No	0	
		Don't know/Don't answer	99	
E10	Are you willing to pay 60,000VND for one HIV test like the one that you have taken today?	Yes	1	
		No	0	0 →**E13**
E11	Are you willing to pay 90,000VND for one HIV test like the one that you have taken today?	Yes	1	
		No	0	**0→E14**
E12	Are you willing to pay 120,000VND for one HIV test like the one that you have taken today?	Yes	1	**1→E14**
		No	0	**0→E14**
E13	Are you willing to pay 30,000VND for one HIV test like the one that you have taken today?	Yes	1	
		No	0	
E14	What is the maximum price are you willing to pay for one HIV test like the one that you have taken today	Amount:______________________		
E15	How frequently do you plan to test for HIV in the coming	Less than once a year	1	
		Once a year	2	

#	Question	Answer	Code	Notes
	time?<br><br>Show or read out loud the answers	Twice a year	3	
		Three times a year	4	
		Four or more times a year	5	
		Won't have HIV test anymore/ Don't know/ Don't answer	99	

THANK YOU!
