# Supplementary material for: Lay provider HIV testing: A promising strategy to reach the undiagnosed key populations in Vietnam
Source: PLoS One. 2018 Dec 31;13(12):e0210063. doi: 10.1371/journal.pone.0210063 (PMC6312239; doi:10.1371/journal.pone.0210063)
Supplement: S2 Text — (PDF) [file pone.0210063.s004.pdf]

BỘ CÂU HỎI VỀ TƯ VẤN XÉT NGHIỆM HIV KHÔNG CHUYÊN

1. Ngày xét nghiệm (Ngày/ tháng/ năm): |_|_| / |_|_| / |_|_|_|_|
2. Mã số của xét nghiệm viên không chuyên: |_|_|_|_|_|_|
3. Mã số của khách hàng: |_|_|_|_|_|_|_|_|_|_|
4. Tỉnh/ thành phố:
   1. Điện Biên 2. Nghệ An 3. Hà Nội 4. Hồ Chí Minh
5. Quận/ Huyện thực hiện quan sát :……………………………………
6. Mã số giám sát viên: ……………………………..

PHẦN A: SÀNG LỌC

STT	Câu hỏi	Trả lời	Mã số	Ghi chú
A1	Anh/ chị đã từng làm xét nghiệm HIV chưa?	Đã từng	1	
		Chưa từng làm xét nghiệm	0	0→ Câu B1
A2	Trong 12 tháng qua, anh/ chị có xét nghiệm HIV không?	Có	1	
		Không	0	
A3	Lần xét nghiệm gần đây nhất, anh/ chị đã làm xét nghiệm ở đâu?	Cơ sở tư vấn và xét nghiệm HIV	1	
		Bệnh viện/ phòng khám nhà nước	2	
		Bệnh viện/ phòng khám tư nhân	3	
		Trung tâm giáo dục lao động xã hội (05-06)	4	
		Xét nghiệm do nhân viên y tế thôn bản hoặc nhân viên cộng đồng/nhân viên CBO	5	Cảm ơn và **dừng cuộc phỏng vấn**
		Tự xét nghiệm	6	
		Xét nghiệm lưu động	7	
		Khác_______________	88	
		Không nhớ/ Không biết	99	
A4	Xin phép anh/ chị có thể chia sẻ với cá nhân tôi về kết quả của xét nghiệm lần gần đây nhất của bản thân mình không?	Dương tính	1	Cảm ơn và **dừng cuộc phỏng vấn,** giới thiệu tư vấn viên để chuyển gửi chăm sóc, điều trị
		Âm tính	2	

	Anh/ chị có quyền từ chối trả lời câu hỏi này nếu như không muốn trả lời	Không xác định	3	
		Không biết/Không trả lời	99	

PHẦN B: THÔNG TIN NHÂN KHẨU

STT	Câu hỏi	Trả lời	Mã số	Ghi chú
B1	Giới tính tự nhận của anh/chị là gì?	Nam	1	
		Nữ	2	
		Chuyển giới nam sang nữ	3	
		Chuyển giới nữ sang nam	4	
B2	Anh/ chị sinh năm nào? *(Năm dương lịch)*	Năm	________	
B3	Anh/ chị là người dân tộc gì?	Kinh	1	
		Thái	2	
		Khơ Mú	3	
		Mông	4	
		Dao	5	
		Hoa	6	
		Khơ Me	7	
		Khác (ghi rõ)_______________	88	
B4	Xin anh/chị cho biết trình độ **học vấn cao nhất** của anh/chị?	Mù chữ	1	
		Tiểu học (1-5)	2	
		Cấp II (6-9)	3	
		Cấp III (10-12)	4	
		Trung cấp, cao đẳng, đại học, sau đại học	5	
B5	Anh/ chị đang làm nghề gì là chính?<br><br>*(nghề nghiệp chính là nghề chiếm nhiều thời gian nhất và đem lại nguồn thu cho bản thân)*	Nông dân, ngư dân, lâm nghiệp	1	
		Công nhân xây dựng/công nhân nhà -máy	2	
		Cán bộ văn phòng/chuyên môn	3	
		Nhân viên dịch vụ giải trí/du lịch	4	
		Doanh nhân/Kinh doanh/Buôn bán	5	
		Sinh viên	6	
		Bán dâm	7	
		Lao động tự do/dịch vụ tự do	8	

STT	Câu hỏi	Trả lời	Mã số	Ghi chú
		Thất nghiệp	9	
		Khác (nêu cụ thể)___________	88	
B6	Trong năm vừa qua, thu nhập trung bình hàng tháng của anh/ chị là bao nhiêu?<br><br>*(Bao gồm tất cả các nguồn thu)*	Số tiền ________________		
		Không trả lời	99	
B7	Tình trạng hôn nhân hiện nay của anh/ chị là gì?	Độc thân (chưa lập gia đình)	1	
		Đã kết hôn	2	
		Đang sống chung với bạn tình nhưng không kết hôn	3	
		Ly thân/ ly dị/ góa	4	
		Khác (ghi rõ)_____________	88	
B8	Anh/chị đã bao giờ quan hệ tình dục để đổi lấy tiền hoặc các tài sản khác chưa?	Có	1	
		Chưa bao giờ	0	
		Không trả lời	99	
B9	Anh đã bao giờ có quan hệ tình dục đường hậu môn với một người nam giới khác chưa?	Không phù hợp (Do khách hàng là NỮ hoặc CHUYỂN GIỚI NỮ SANG NAM)	2	
		Có	1	Chỉ hỏi cho nam giới hoặc người chuyển giới từ nam sang nữ
		Chưa bao giờ	0	
		Không trả lời	99	
B10	Anh/chị đã từng tiêm chích một chất gây nghiện (ma túy) nào không?	Có	1	
		Không	0	
		Không trả lời	99	

PHẦN C: CÂU HỎI TRƯỚC XÉT NGHIỆM

STT	Câu hỏi	Trả lời	Mã số	Ghi chú
C1	Trước đây, anh/chị **đã từng nghe nói** về xét nghiệm HIV do người không phải là cán bộ y tế thực hiện chưa, ví dụ như nhân viên cộng đồng/nhân viên CBO hoặc y tế thôn bản ?	Có	1	0 → Câu C3
		Không	0	
		Không nhớ	99	99→ Câu C3
C2	Nếu có, anh/chị biết được thông tin đó từ nguồn nào/từ ai? ***(Câu hỏi nhiều lựa chọn)***	Tiếp cận viên cộng đồng	1	
		Nhân viên y tế	2	
		Bạn bè	3	
		Họ hàng	4	
		Vợ/chồng/bạn tình	5	
		Facebook	6	
		Internet	7	
		TiVi, đài	8	
		Tờ rơi	9	
		Pano, Áp phích, bảng thông tin	10	
		Cuộc họp, buổi truyền thông cộng đồng	11	
		Khác (ghi rõ)	88	
C3	Vì lý do gì mà Anh/chị lựa chọn làm xét nghiệm do người không phải là cán bộ y tế thực hiện (nhân viên cộng đồng/nhân viên CBO hoặc y tế thôn bản)? ***(Câu hỏi nhiều lựa chọn)***	Không lộ danh tính	1	
		Đảm bảo bí mật	2	
		Đảm bảo riêng tư	3	
		Thủ tục đơn giản	4	
		Địa điểm gần, đi lại thuận tiện	5	
		Thời gian linh hoạt,	6	
		Biết kết quả nhanh	7	
		Giá phải chăng	8	
		Miễn phí	9	
		Được tư vấn cụ thể, rõ ràng	10	
		Nhân viên thân thiện, chu đáo	11	
		Xét nghiệm chính xác	12	
		Được chỉ dẫn, hỗ trợ kết nối với cơ sở y tế	13	

STT	Câu hỏi	Trả lời	Mã số	Ghi chú
		Được tiếp cận viên cộng đồng giới thiệu	14	
		Được nhân viên y tế giới thiệu	15	
		Khác (cụ thể)______________	88	
C4	Để anh/chị quyết định làm xét nghiệm này anh/chị cần biết những thông tin gì nhất?<br><br>(chọn 3 ý quan trọng nhất) – Cho xem đáp án để chọn.	Giá thành của xét nghiệm	1	
		Độ chính xác của xét nghiệm	2	
		Tính bảo mật của xét nghiệm	3	
		Thời gian làm xét nghiệm và trả kết quả	4	
		Qui trình làm xét nghiệm	5	
		Nơi thực hiện xét nghiệm ở đâu/ như thế nào	6	
		Trang thiết bị, dụng cụ xét nghiệm	7	
		Nhân viên xét nghiệm là ai/trình độ thế nào	8	
		Thái độ phục vụ, đón tiếp	9	
		Chất lượng tư vấn/khả năng thuyết phục của nhân viên tư vấn	10	
		Có thể kết nối với các dịch vụ khác như xét nghiệm khẳng định, chăm sóc và điều trị HIV	11	
		Khác (ghi rõ)______________	88	
C5	Anh/chị muốn nhận các thông tin nêu trên từ ai/từ nguồn nào nhất?<br><br>(chọn 3 ý quan trọng nhất) – Cho xem đáp án để chọn.	Tiếp cận viên cộng đồng	1	
		Nhân viên y tế	2	
		Bạn bè	3	
		Họ hàng	4	
		Vợ/chồng/bạn tình	5	
		Facebook	6	
		Internet	7	
		TiVi, đài	8	
		Tờ rơi	9	
		Pano, Áp phích, bảng thông tin	10	
		Cuộc họp, buổi truyền thông cộng đồng	11	
		Khác (ghi rõ)	88	

SAU KHI KẾT THÚC PHẦN C, NHÂN VIÊN XÉT NGHIỆM KHÔNG CHUYÊN SẼ BẮT ĐẦU QUI TRÌNH TƯ VẤN TRƯỚC XÉT NGHIỆM và LÀM XÉT NGHIỆM

QUAN SÁT VIÊN TIẾN HÀNH QUY TRÌNH QUAN SÁT

(Đánh dấu X vào cột phù hợp)

PHẦN D1. QUAN SÁT TRƯỚC XÉT NGHIỆM

STT	Nội dung	Không	Có	Đạt chuẩn	Ghi chú
	**CHUẨN BỊ DỤNG CỤ**				
D1	Que xét nghiệm Determine HIV 1/2			Không điền	
D2	Kim chích máu			Không điền	
D3	Dung dịch đệm			Không điền	
D4	Ống mao dẫn			Không điền	
D5	Miếng bông sát khuẩn			Không điền	
D6	Miếng bông khô			Không điền	
D7	Băng dính cá nhân			Không điền	
D8	Găng tay			Không điền	
D9	Khay đựng dụng cụ			Không điền	
D10	3 túi đựng rác riêng biệt			Không điền	
D11	Đồng hồ đếm giờ (bất cứ đồng hồ nào)			Không điền	
	**TƯ VẤN TRƯỚC XÉT NGHIỆM HIV, TRONG ĐÓ NHẤN MẠNH:**				
D12	Xét nghiệm là hoàn toàn tự nguyện			Không điền	
D13	Mọi thông tin liên quan đều được giữ bí mật, riêng tư.			Không điền	
D14	Giải thích quy trình xét nghiệm (chích máu đầu ngón tay, lấy máu vào ống mao dẫn, nhỏ máu vào que thử, chờ và đọc kết quả)			Không điền	
D15	Giải thích ý nghĩa của kết quả xét nghiệm : *Xét nghiệm này là chính xác nhưng chỉ là xét nghiệm sàng lọc để phân loại ban đầu, chưa có ý nghĩa khẳng định.*			Không điền	
D16	Khẳng định khách hàng đồng ý tham gia xét nghiệm (**Đạt chuẩn:** khách hàng ký phiếu tham gia xét nghiệm hoặc thỏa thuận				

STT	Nội dung	Không	Có	Đạt chuẩn	Ghi chú
	miệng)				

PHẦN D2. QUAN SÁT THỰC HIỆN XÉT NGHIỆM

STT	Nội dung	Có	Không	Đạt chuẩn	Ghi chú
**THỰC HIỆN XÉT NGHIỆM**					
D17	Ghi mã số khách hàng lên que thử<br>**(Đạt chuẩn:** mã khách hàng ghi trên que thử trùng với mã khách hàng trong sổ của xét nghiệm viên)				Mã KH có 4 kí tự số
D18	Người làm xét nghiệm rửa tay (với nước thường hoặc nước rửa tay dự án cung cấp)			Không điền	
D19	Người làm xét nghiệm đi găng tay			Không điền	
D20	Xé vỏ que thử<br>(**Đạt chuẩn:** Không chạm tay vào vùng nhỏ mẫu của que thử)				
D21	Xoa nhẹ ngón tay từ gốc đến đốt 2 *(ngón giữa hoặc áp út)*			Không điền	
D22	Sát khuẩn đầu ngón tay bằng miếng sát khuẩn			Không điền	
D23	Chích máu đầu ngón tay<br>(khoanh tròn vào số tương ứng)	1. Một lần<br>2. Hai lần<br>3. Ba lần<br>4. Ba lần nhưng không lấy được máu			**Nếu chọn “đáp án 4” -> kết thúc quan sát**
D24	Dùng miếng bông vô khuẩn lau bỏ giọt máu đầu			Không điền	

STT	Nội dung	Có	Không	Đạt chuẩn	Ghi chú
D25	Dùng ống mao dẫn lấy máu xét nghiệm<br>**(Đạt chuẩn:** lấy máu đến giữa hai vạch đen của ống mao dẫn và không có quá nhiều bọt khí)				
D26	Cầm máu đầu ngón tay			Không điền	
D27	Đưa mẫu máu vào vùng nhỏ mẫu của que thử<br>**(Đạt chuẩn:** Đảm bảo máu thấm hết vào vùng nhỏ mẫu của que thử)				
D28	Nhỏ dung dịch đệm vào vùng nhỏ mẫu<br>**(Đạt chuẩn:** Không chạm đầu lọ dung dịch vào mẫu xét nghiệm trên que thử)				
D29	Đọc kết quả đúng thời gian quy định?<br>**(Đạt chuẩn**: trong khoảng 15 – 60 phút, không đọc kết quả sau 60 phút.)				
D30	Đọc đúng kết quả xét nghiệm?<br>**(Đạt chuẩn:**<br>- *Không phản ứng: Que thử xuất hiện 1 vạch đỏ (ô vạch chứng)*<br>- *Có phản ứng: que thử xuất hiện 2 vạch đỏ (ô vạch chứng và ô kết quả)*<br>- *Không đủ tiêu chuẩn: không xuất hiện vạch màu nào hay chỉ xuất hiện vạch bên dưới (vạch ô kết quả)*				Giám sát viên kiểm tra xem đọc có đúng không?
D31	Quan sát viên đọc kết quả của xét nghiệm này là:	1. Không phản ứng<br>2. Có phản ứng<br>3. Không đủ tiêu chuẩn			Giám sát viên khoanh vào số tương ứng

PHẦN D3. QUAN SÁT SAU XÉT NGHIỆM

STT	Nội dung	Có	Không	Đạt chuẩn	Ghi chú
**TƯ VẤN SAU XÉT NGHIỆM**					
D32	Khẳng định lại sự bảo mật về thông tin liên quan đến xét nghiệm và khách hàng			Không	

				điền	
**KẾT QUẢ KHÔNG PHẢN ỨNG**					
D33	Giải thích ý nghĩa kết quả không phản ứng: **(Đạt chuẩn:** *kết quả của bạn là không phản ứng, kết quả này khẳng định từ 3 tháng trở về trước, bạn chưa nhiễm HIV. Kết quả này không phản ánh tình trạng nhiễm HIV của bạn trong 3 tháng gần đây.)*				
D34	Trao đổi về các biện pháp dự phòng HIV (ví dụ sử dụng bao cao su, bơm kim tiêm sạch, điều trị methadone).			Không điền	
D35	Hẹn lịch xét nghiệm lại **(Đạt chuẩn:** xét nghiệm lại sau 3 tháng (nếu khách hàng có hành vi nguy cơ/phơi nhiễm trong 3 tháng qua) hoặc định kỳ 6 tháng 1 lần đối với các nhóm có nguy cơ cao.)				
**KẾT QUẢ CÓ PHẢN ỨNG**					
D36	Giải thích ý nghĩa kết quả có phản ứng **(Đạt chuẩn:** *kết quả của bạn là có phản ứng. Tuy nhiên, đây chỉ mới là xét nghiệm phân loại ban đầu, chưa thể khẳng định chắc chắn. Việc cần làm là bạn nhanh chóng đến cơ sở y tế làm thêm xét nghiệm để khẳng định tình trạng nhiễm HIV.)*				
D37	Trao đổi về việc cần thiết làm xét nghiệm chẩn đoán			Không điền	
D38	Giới thiệu khách hàng đến CSYT để lấy máu			Không điền	
D39	Thể hiện sự đồng cảm và sẵn sàng hỗ trợ khách hàng đi làm xét nghiệm khẳng định **(Đạt chuẩn:** viết phiếu chuyển gửi cho khách hàng, thu xếp lịch hẹn với cơ sở y tế cho khách hàng, và đề xuất đi cùng khách hàng đến cơ sở y tế nếu họ đồng ý.)				
**KẾT QUẢ KHÔNG ĐỦ TIÊU CHUẨN**					
D40	Giải thích lý do và xin lỗi khách hàng về sự bất tiện khi phải thực hiện lại xét nghiệm			Không điền	
D41	Xin phép thực hiện lại			Không điền	
D42	Khuyến khích khách hàng đưa bạn tình đi xét nghiệm			Không điền	
**HOÀN THIỆN**					
D43	Thu dọn rác thải vào túi rác thải y tế mang về trạm y tế để xử lý theo quy định				

	**(Đạt chuẩn:** rác thải được bỏ vào 3 túi nilon riêng biệt/3 thùng rác riêng biệt: 1. Que thử; 2. Kim chích máu; 3.Rác)				
D44	Rửa tay			Không điền	
D45	Ghi chép thông tin khách hàng vào sổ			Không điền	

Lưu ý: Đánh dấu X vào cả 2 cột "**Có**" và "**Đạt chuẩn**" nếu câu nào phù hợp với "**Đạt chuẩn:...**"

PHẦN E: CÂU HỎI SAU XÉT NGHIỆM (PHỎNG VẤN RIÊNG KHÁCH HÀNG)

STT	Câu hỏi	Trả lời	Mã số	Ghi chú
E1	Nhìn chung, anh/ chị có **hài lòng** với dịch vụ xét nghiệm mà anh/chị vừa nhận không? (Cho xem hoặc đọc đáp án)	Rất hài lòng	1	
		Hài lòng	2	
		Bình thường	3	**3** → Câu E4
		Không hài lòng	4	**4** → Câu E3
		Rất không hài lòng	5	5 → Câu E3
E2	Nếu hài lòng, anh/chị thấy hài lòng về những điều gì?<br><br>***(Chọn nhiều đáp án)***	Địa điểm xét nghiệm phù hợp (thuận tiện/sạch sẽ/kín đáo)	1	**Kết thúc E2 sẽ chuyển sang E4**
		Thời gian linh hoạt, ít chờ đợi, kết quả nhanh	2	
		Thái độ nhân viên thân thiện, chu đáo	3	
		Kỹ năng làm xét nghiệm của nhân viên tốt	4	
		Cách lấy mẫu xét nghiệm đơn giản, không đau (chích đầu ngón tay/lấy nước bọt)	5	
		Tư vấn đầy đủ, dễ hiểu	6	
		Đảm bảo bí mật của xét nghiệm	7	
		Miễn phí	8	
		Giá cả hợp lý	9	
		Được chỉ dẫn, kết nối với các dịch vụ khác như xét nghiệm khẳng định, chăm sóc và điều trị HIV	10	

STT	Câu hỏi	Trả lời	Mã số	Ghi chú
		Khác (ghi rõ)___________	88	
E3	Nếu KHÔNG hài lòng, anh/chị thấy KHÔNG hài lòng về những điều gì?<br><br>***(Chọn nhiều đáp án)***	Địa điểm xét nghiệm không phù hợp (đi xa/không sạch sẽ/không kín đáo)	1	
		Lịch làm xét nghiệm không phù hợp, chờ đợi lâu	2	
		Thái độ nhân viên không phù hợp (thiếu cởi mở, thân thiện)	3	
		Kỹ năng làm xét nghiệm chưa đảm bảo	4	
		Tư vấn vội vàng, chưa đầy đủ	5	
		Tính bảo mật và riêng tư chưa tốt	6	
		Chi phí tốn kém khi làm xét nghiệm	7	
		Chưa kết nối với các dịch vụ khác như như xét nghiệm khẳng định, chăm sóc và điều trị HIV	8	
		Kết quả xét nghiệm không rõ ràng	9	
		Khác (ghi rõ)___________	88	
E4	Anh/ chị muốn được nhân viên cộng đồng/nhân viên CBO hoặc y tế thôn bản thực hiện xét nghiệm HIV **ở đâu nhất**?<br><br>*Cho xem hoặc đọc đáp án*<br><br>*Chọn một đáp án*	Tại nhà của anh/chị	1	
		Tại nơi làm việc của anh/chị	2	
		Tại nhà của nhân viên xét nghiệm	3	
		Tại nơi làm viêc của nhân viên xét nghiệm (ví dụ văn phòng CBO)	4	
		Tại trạm y tế xã phường	5	
		Ở một nơi kín đáo, không thuộc các địa điểm nêu trên	6	
		Không quan tâm, ở đâu cũng được	7	
		Khác (ghi rõ) ____________	88	
E5	Nếu tiếp tục làm xét nghiệm HIV tại cộng đồng, anh/chị thích ai làm xét nghiệm cho anh/chị **nhất**?	Nhân viên cộng đồng/nhân viên CBO	1	
		Y tế thôn bản	2	

STT	Câu hỏi	Trả lời	Mã số	Ghi chú
	Cho xem hoặc đọc đáp án<br><br>***Chọn một đáp án***	Cán bộ Y tế xã	3	
		Cán bộ y tế y tế huyện làm xét nghiệm lưu động	4	
		Tôi sẽ tự làm xét nghiệm	5	
		Khác (ghi rõ)……………………	88	
		Sẽ không làm xét nghiệm nữa/Không biết/không trả lời	99	
E6	Nếu tiếp tục làm xét nghiệm HIV, **hình thức lấy mẫu** xét nghiệm nào sau đây anh/ chị **thích nhất**?<br><br>*Cho xem hoặc đọc đáp án*<br><br>(Giải thích sự khác nhau giữa 3 hình thức và cho xem tranh ảnh)<br><br>*Chọn một đáp án*	Lấy mẫu nước bọt bằng tăm bông	1	
		Chích máu đầu ngón tay	2	
		Lấy máu tĩnh mạch	3	
		Hình thức nào cũng được	4	
		Sẽ không làm xét nghiệm HIV nữa/Không biết/không trả lời	99	
E7	Nếu anh/ chị được hướng dẫn cách tự làm xét nghiệm HIV cho mình (tự lấy máu đầu ngón tay hoặc lấy nước bọt bằng tăm bông, tự nhỏ dung dịch lên que thử và tự đọc kết quả*)*, anh/ chị có muốn tự làm không?	Có	1	
		Không	2	
		Thế nào cũng được	3	
		Không biết/ không trả lời	4	
E8	Hôm nay Anh/ chị đã được làm xét nghiệm HIV tại cộng đồng, vậy anh/ chị thích làm xét nghiệm HIV ở đâu nhất trong các phương án sau?<br><br>*Cho xem hoặc đọc đáp án*<br><br>***(Chọn một đáp án)***	Xét nghiệm ở cộng đồng giống buổi hôm nay	1	
		Xét nghiệm ở một nơi cố định, ví dụ như trung tâm xét nghiệm và tư vấn của nhà nước	2	
		Xét nghiệm ở phòng khám hay bệnh viện tư nhân	3	
		Chỗ nào cũng được	4	

STT	Câu hỏi	Trả lời	Mã số	Ghi chú
		Không biết	5	
E9	Anh/ chị có muốn **giới thiệu** dịch vụ xét nghiệm mà hôm nay anh/chị vừa được làm với người khác không (ví dụ như: vợ/chồng/bạn tình, bạn bè hoặc người thân trong gia đình)?	Có	1	
		Không	0	
		Không biết/không trả lời	99	
E10	Anh/chị có sẵn sàng trả tiền cho 1 lần làm xét nghiệm HIV như hôm nay với giá 60.000 đồng?	Có	1	
		Không	0	**0→E13**
E11	Anh/chị có sẵn sàng trả tiền cho 1 lần làm xét nghiệm HIV như hôm nay với giá 90.000 đồng?	Có	1	
		Không	0	**0→E14**
E12	Anh/chị có sẵn sàng trả tiền cho 1 lần làm xét nghiệm HIV như hôm nay với giá 120.000 đồng?	Có	1	1→**E14**
		Không	0	**0 →E14**
E13	Anh/chị có sẵn sàng trả tiền cho 1 lần làm xét nghiệm HIV như hôm nay với giá 30.000 đồng?	Có	1	
		Không	0	
E14	Mức giá tối đa anh/chị sẵn sàng chi trả cho 1 lần làm xét nghiệm HIV như hôm nay là bao nhiêu?	Ghi nguyên giá tiền	------	
E15	Trong thời gian tới, anh/ chị dự định bao lâu đi xét nghiệm HIV một lần?<br><br>*Cho xem hoặc đọc đáp án*	Vài năm làm 1 lần	1	
		1 lần 1 năm	2	
		2 lần 1 năm	3	
		3 lần 1 năm	4	
		4 lần 1 năm hoặc nhiều hơn	5	
		Sẽ không xét nghiệm HIV nữa/ Không biết/ Không trả lời	99	

XIN CẢM ƠN!
